# Draft genome sequence of marine alphaproteobacterial strain HIMB11, the first cultivated representative of a unique lineage within the *Roseobacter* clade possessing an unusually small genome

**DOI:** 10.4056/sigs.4998989

**Published:** 2014-03-15

**Authors:** Bryndan P. Durham, Jana Grote, Kerry A. Whittaker, Sara J. Bender, Haiwei Luo, Sharon L. Grim, Julia M. Brown, John R. Casey, Antony Dron, Lennin Florez-Leiva, Andreas Krupke, Catherine M. Luria, Aric H. Mine, Olivia D. Nigro, Santhiska Pather, Agathe Talarmin, Emma K. Wear, Thomas S. Weber, Jesse M. Wilson, Matthew J. Church, Edward F. DeLong, David M. Karl, Grieg F. Steward, John M. Eppley, Nikos C. Kyrpides, Stephan Schuster, Michael S. Rappé

**Affiliations:** 1 Center for Microbial Oceanography: Research and Education, University of Hawaii, Honolulu, Hawaii, USA; 2 Department of Microbiology, University of Georgia, Athens, Georgia, USA; 3 Department of Oceanography, University of Hawaii, Honolulu, Hawaii, USA; 4 Graduate School of Oceanography, University of Rhode Island, Narragansett, Rhode Island, USA; 5 School of Oceanography, University of Washington, Seattle, Washington, USA; 6 Department of Marine Sciences, University of Georgia, Athens, Georgia, USA; 7 Marine Biological Laboratory, Woods Hole, Massachusetts, USA; 8 Department of Microbiology, Cornell University, Ithaca, New York, USA; 9 Observatoire Océanologique de Villefranche, Villefranche-sur-mer, France; 10 Universidad Del Magdalena, Santa Marta, Colombia; 11 Max Plank Institute for Marine Microbiology, Bremen, Germany; 12 Department of Ecology and Evolutionary Biology, Brown University, Providence, Rhode Island, USA; 13 Department of Geophysical Sciences, University of Chicago, Chicago, Illinois, USA; 14 School for Marine Science and Technology, University of Massachusetts Dartmouth, Dartmouth, Massachusetts, USA; 15 Department of Earth System Science, University of California Irvine, Irvine, CA, USA; 16 Marine Science Institute, University of California Santa Barbara, Santa Barbara, California, USA; 17 Department of Atmospheric and Ocean Sciences, University of California Los Angeles, Los Angeles, California, USA; 18 School of Natural Sciences, University of California Merced, Merced, California, USA; 19 Department of Civil and Environmental Engineering, Massachusetts Institute of Technology, Cambridge, Massachusetts, USA; 20 Department of Energy Joint Genome Institute, Walnut Creek, California, USA; 21 Singapore Centre on Environmental Life Sciences Engineering, Singapore; 22 Hawaii Institute of Marine Biology, University of Hawaii, Kaneohe, Hawaii, USA

**Keywords:** marine bacterioplankton, *Roseobacter*, aerobic anoxygenic phototroph, dimethylsulfoniopropionate

## Abstract

Strain HIMB11 is a planktonic marine bacterium isolated from coastal seawater in Kaneohe Bay, Oahu, Hawaii belonging to the ubiquitous and versatile *Roseobacter* clade of the alphaproteobacterial family *Rhodobacteraceae*. Here we describe the preliminary characteristics of strain HIMB11, including annotation of the draft genome sequence and comparative genomic analysis with other members of the *Roseobacter* lineage. The 3,098,747 bp draft genome is arranged in 34 contigs and contains 3,183 protein-coding genes and 54 RNA genes. Phylogenomic and 16S rRNA gene analyses indicate that HIMB11 represents a unique sublineage within the *Roseobacter* clade. Comparison with other publicly available genome sequences from members of the *Roseobacter* lineage reveals that strain HIMB11 has the genomic potential to utilize a wide variety of energy sources (e.g. organic matter, reduced inorganic sulfur, light, carbon monoxide), while possessing a reduced number of substrate transporters.

## Introduction

Bacteria belonging to the *Roseobacter* lineage of marine *Alphaproteobacteria* account for a substantial fraction (ranging ~10 – 25%) of bacterioplankton cells in surface ocean seawater [1-4], making them one of a relatively small number of suitable targets for scientists investigating the ecology of abundant marine bacterial groups. Focused genome sequencing efforts have provided significant insights into the functional and ecological roles for this group [5-7]. In 2004, the first member of this group to have its genome sequenced, *Ruegeria pomeroyi* (basonym *Silicibacter pomeroyi*) strain DSS-3 [8], revealed strategies used by the *Roseobacter* group for nutrient acquisition in the marine environment. To date, over 40 genomes have been sequenced from members of the *Roseobacter* lineage. Comparative analysis among 32 of these genomes indicates that members of this group are ecological generalists, having relatively plastic requirements for carbon and energy metabolism, which may allow them to respond to a diverse range of environmental conditions [9]. For example, members of the *Roseobacter* lineage have the genomic potential to obtain energy via oxidation of organic substrates, oxidation of inorganic compounds, and/or sunlight-driven electron transfer via bacteriochlorophyll *a*, proteorhodopsin, or xanthorhodopsin phototrophic systems. Genome analyses as well as culture experiments have also revealed a variety of mechanisms by which roseobacters may associate and interact with phytoplankton and other eukaryotes. These include genes involved in uptake of compounds produced by algae such as peptides, amino acids, putrescine, spermidine, and DMSP [10,11], as well as genes for chemotaxis, attachment, and secretion [12].

Strain HIMB11 was isolated from surface seawater collected from Kaneohe Bay off the coast of Oahu, Hawaii, USA in May, 2005. Subsequent 16S rRNA gene sequence comparisons revealed it to be a member of the *Roseobacter* clade of marine bacterioplankton [13] that was highly abundant after a storm-induced phytoplankton bloom in the bay [14]. Here, we present a preliminary set of features for strain HIMB11, a description of the draft genome sequence and annotation, and a comparative analysis with 35 other genome sequences from members of the *Roseobacter* lineage. Genome annotation revealed strain HIMB11 to have the genetic potential for bacteriochlorophyll-based aerobic anoxygenic phototrophic (AAnP) metabolism and degradation of the algal-derived compound DMSP along with production of the climate-relevant gas dimethylsulfide (DMS), and oxidation of the greenhouse gas carbon monoxide (CO). Collectively, these features indicate the potential for strain HIMB11 to participate in the biogeochemical cycling of sulfur and carbon, and concomitantly affect global climate processes.

### Classification and features

Strain HIMB11 was isolated by a high-throughput, dilution-to-extinction approach [15] from surface seawater collected near the coast of Oahu, Hawaii, USA, in the tropical North Pacific Ocean. The strain was isolated in seawater sterilized by tangential flow filtration and amended with low concentrations of inorganic nitrogen and phosphorus (1.0 µM NH_4_Cl, 1.0 µM NaNO_3_, and 0.1 µM KH_2_PO_4_).

**Figure 1:**
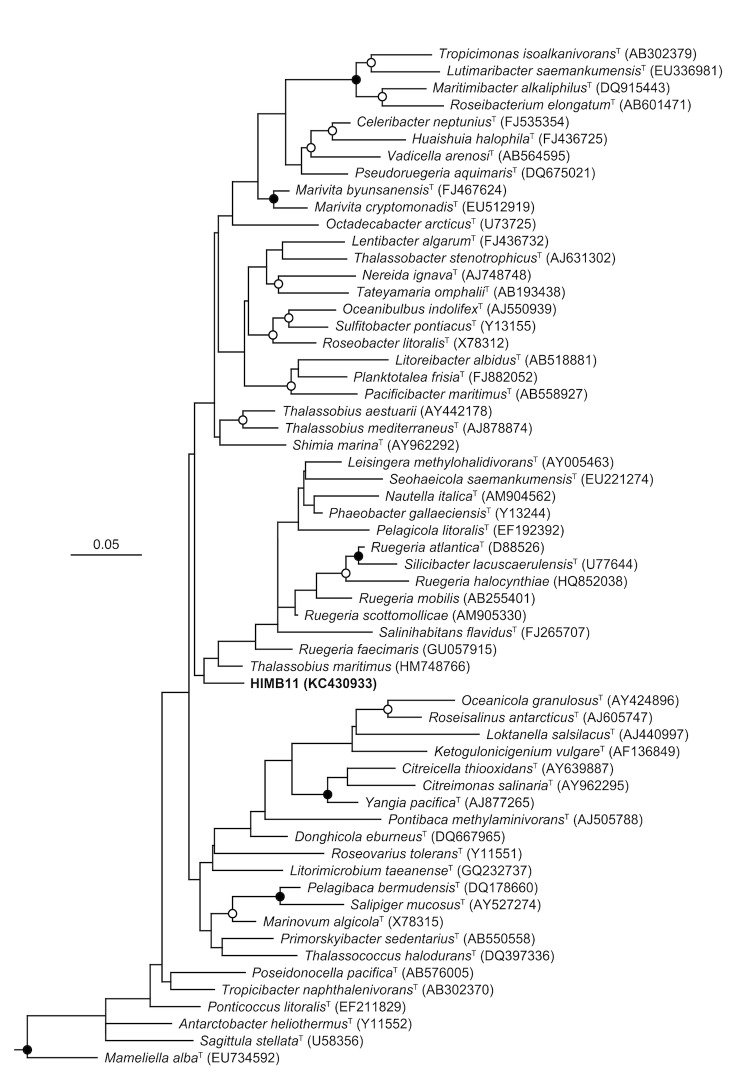
Phylogenetic relationships between HIMB11 and bacterial strains belonging to the *Roseobacter* clade. SSU rRNA gene sequences were aligned with version 111 of the ‘All-Species Living Tree’ project SSU rRNA gene database [16] using the ARB software package [17]. The phylogeny was constructed from nearly full-length gene sequences using the RAxML maximum likelihood method [18] within ARB, filtered to exclude alignment positions that contained gaps or ambiguous nucleotides in any of the sequences included in the tree. Bootstrap analyses were determined by RAxML [19] via the raxmlGUI graphical front end [20]. The scale bar corresponds to 0.05 substitutions per nucleotide position. Open circles indicate nodes with bootstrap support between 50-80%, while closed circles indicate bootstrap support >80%, from 500 replicates. A variety of *Archaea* were used as outgroups.

Comparative analysis of the HIMB11 16S rRNA gene sequence to those from cultured, sequenced roseobacters indicates that HIMB11 occupies a unique lineage that is divergent from the 16S rRNA gene sequences of *Roseobacter* strains already in culture (Figure 1). Based on the National Center for Biotechnology Information (NCBI) non-redundant database, the HIMB11 16S rRNA gene sequence is most similar (~99% nucleotide identity) to a large number of environmental gene clones obtained from various marine environments that exclusively fall in the *Roseobacter* lineage of *Alphaproteobacteria*.

**Figure 2:**
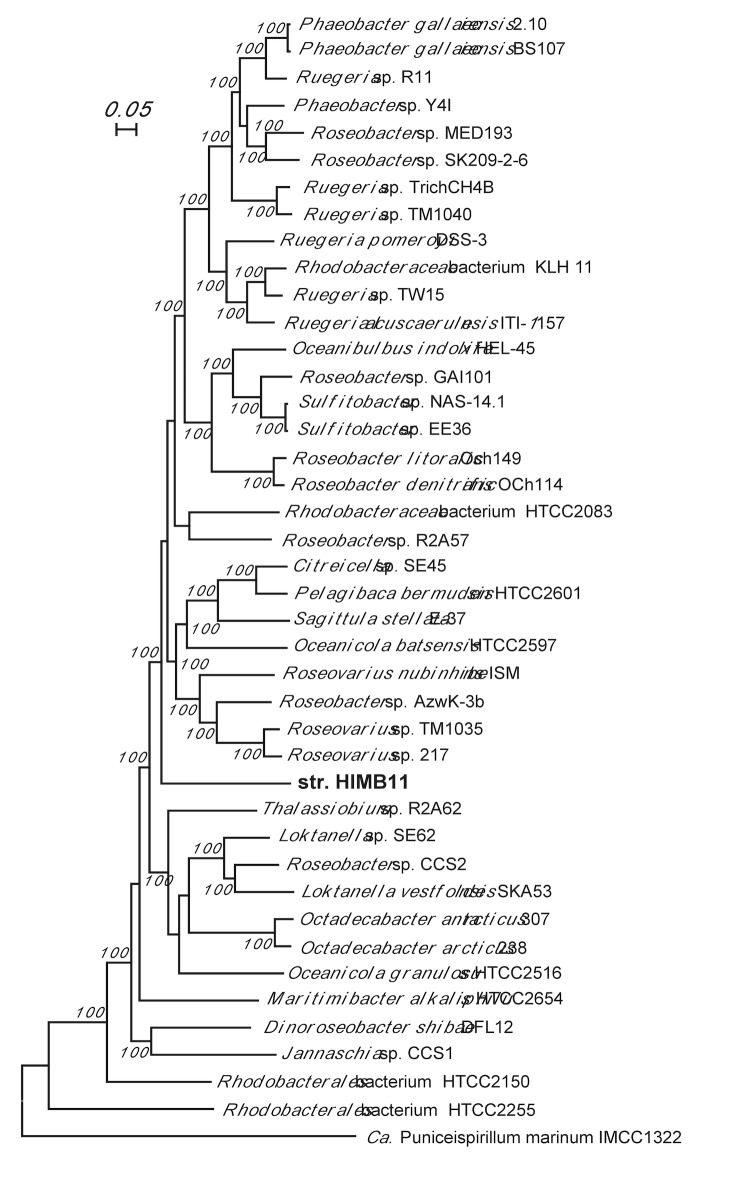
Maximum likelihood phylogenomic tree derived from a concatenation of 719 shared single-copy orthologous protein sequences from strain HIMB11, 40 other *Roseobacter* members, and the SAR116 clade member *Ca. Puniceispirillum marinum* IMCC1322 (outgroup) using the RAxML maximum likelihood method [18]. After a reciprocal BLAST search [22] was performed for all possible genome pairs at the amino acid level using an E value of 10^-6^, orthologous genes in each of the genome pairs were identified using the MSOAR software [23], which assigns orthologs by considering both sequence similarity and gene context information. One genome was picked at random as the reference genome, and pairwise orthologs were linked to the reference. Values at the nodes show the number of times the clade defined by that node appeared in the 100 bootstrapped data sets. The scale bar indicates number of substitutions per site. Bootstrap values lower than 50% were not displayed.

Because of the significant sequence variation in 16S rRNA genes (up to 11%) and the prevalence of horizontal gene transfer within the clade, establishing a taxonomic framework for roseobacters remains a challenge [9]. When genome sequence data is available, it is often more informative to perform a phylogenomic analysis based on shared orthologs versus 16S rRNA phylogenetic analysis alone [9,21]. A maximum likelihood tree constructed using 719 shared orthologous protein sequences supported the 16S rRNA gene-based analysis by revealing that HIMB11 formed a unique sublineage of the *Roseobacter* clade (Figure 2).

**Figure 3:**
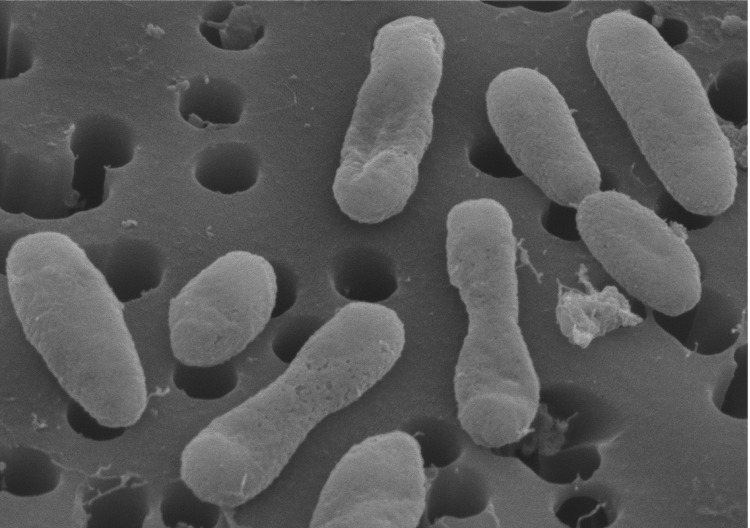
Scanning electron micrograph of strain HIMB11. For scale, the membrane pore size is 0.2 μm in diameter.

HIMB11 cells are short, irregular rods (0.3-0.5 x 0.8 µm) that are generally smaller in size than previously reported for other cultured *Roseobacter* strains (e.g. described taxa in Bergey’s Manual range from 0.5-1.6 – 1.0-4.0 µm) [24] (Figure 3). HIMB11 is likely motile, as the genes necessary to build flagella are present (e.g. *fli*, *flg*). Based on the ability of HIMB11 to grow in dark or light on a medium consisting solely of sterile seawater amended with inorganic nitrogen and phosphorus, and the absence any of the known pathways for inorganic carbon fixation, the strain is presumed to acquire carbon and energy via the oxidation of components of the dissolved organic carbon pool in natural seawater. Based on the presence of carbon-monoxide-oxidizing genes (i.e. *cox*L, forms I and II) [25,26] as well as bacteriochlorophyll-based phototrophy genes (e.g. *puf*, *puh*, *bch*) [27,28], HIMB11 is hypothesized to oxidize both organic and inorganic compounds as well as obtain energy from light [5]. A summary of these and other features is shown in Table 1.

**Table 1:** Classification and general features of strain HIMB11 according to the MIGS recommendations [29].

**MIGS ID**	**Property**	**Term**	**Evidence code**^a^
	Current classification	Domain *Bacteria*	TAS [30]
		Phylum *Proteobacteria*	TAS [31]
		Class *Alphaproteobacteria*	TAS [32,33]
		Order *Rhodobacterales*	TAS [32,34]
		Family *Rhodobacteraceae*	TAS [32,35]
		Genus not assigned	
		Species not assigned	
		Strain HIMB11	
	Gram stain	Negative	NAS
	Cell shape	Short irregular rods	IDA
	Motility	Flagella	NAS
	Sporulation	Non-sporulating	NAS
	Temperature range	Mesophile	IDA
	Optimum temperature	Unknown	
	Carbon source	Ambient seawater DOC	TAS [36]
	Energy source	Mixotrophic	NAS
	Terminal electron receptor		
MIGS-6	Habitat	Seawater	IDA
MIGS-6.3	Salinity	~35.0 ‰	IDA
MIGS-22	Oxygen	Aerobic	NAS
MIGS-15	Biotic relationship	Free-living	TAS [36]
MIGS-14	Pathogenicity	None	NAS
MIGS-4	Geographic location	Kaneohe Bay, Hawaii	TAS [14]
MIGS-5	Sample collection time	18 May, 2005	TAS [14]
MIGS-4.1	Latitude - Longitude	21.44, -157.78	TAS [14]
MIGS-4.2			
MIGS-4.3	Depth	~1 m	TAS [14]
MIGS-4.4	Altitude		

a) Evidence codes - IDA: Inferred from Direct Assay; TAS: Traceable Author Statement (i.e., a direct report exists in the literature); NAS: Non-traceable Author Statement (i.e., not directly observed for the living, isolated sample, but based on a generally accepted property for the species, or anecdotal evidence). These evidence codes are from the Gene Ontology project [37].

## Genome sequencing information

### Genome project history

The genome of strain HIMB11 was selected for sequencing based on its phylogenetic affiliation with the widespread and ecologically important *Roseobacter* clade of marine bacterioplankton and its periodically high abundance in coastal Hawaii seawater [14]. The genome sequence was completed on May 25, 2011, and presented for public access on September 15, 2013. The genome project is deposited in the Genomes OnLine Database (GOLD) as project Gi09592. The Whole Genome Shotgun project has been deposited at DDBJ/EMBL/GenBank under the accession number AVDB00000000. The version described in this paper is version AVBD01000000. Table 2 presents the main project information and its association with MIGS version 2.0 compliance [29].

**Table 2:** Project information

**MIGS ID**	**Property**	**Term**
MIGS-31	Finishing quality	Draft
MIGS-28	Libraries used	One standard 454 pyrosequence titanium library
MIGS-29	Sequencing platforms	454 GS FLX Titanium
MIGS-31.2	Fold coverage	121× pyrosequence
MIGS-30	Assemblers	Newbler version 2.5.3
MIGS-32	Gene calling method	Prodigal 1.4, GenePRIMP
	Genome Database release	IMG; 2506210027
	Genbank ID	AVDB00000000
	Genbank Date of Release	September 15, 2013
	GOLD ID	Gi09592
	Project relevance	Environmental

### Growth conditions and DNA isolation

Strain HIMB11 was grown at 27 °C in 60 L of coastal Hawaii seawater sterilized by tangential flow filtration and supplemented with 10 µM NH_4_Cl, 1.0 µM KH_2_PO_4_, and 1.0 µM NaNO_3_ (final concentrations). Cells from the liquid culture were collected on a membrane filter, and DNA was isolated using a standard phenol/chloroform/isoamyl alcohol extraction protocol. A total of 50 μg of DNA was obtained.

### Genome sequencing and assembly

The HIMB11 genome was sequenced at the Pennsylvania State University Center for Comparative Genomics and Bioinformatics (University Park, PA, USA) using a 454 GS FLX platform and Titanium chemistry from 454 Life Sciences (Branford, CT, USA). The sequencing library was prepared in accordance with 454 instructions and was carried out on a full 454 picotiter plate. This yielded 1,550,788 reads with an average length of 359 bp, totaling 556,821,617 bp. A subset of 1,336,895 reads was ultimately assembled using the Newbler assembler version 2.5.3, yielding a final draft genome of 34 contigs representing 3,098,747 bp. This provided 121× coverage of the genome.

### Genome annotation

Genes were identified using Prodigal 1.4 [38] as part of the genome annotation pipeline in the Integrated Microbial Genomes Expert Review (IMG-ER) system [39,40] developed by the Joint Genome Institute (Walnut Creek, CA, USA). Predicted coding sequences were translated and used as queries against the NCBI non-redundant database and UniProt, TIGRFam, Pfam, PRIAM, KEGG, COG, and InterPro databases. The tRNAScanSE tool [41] was used to identify tRNA genes, and ribosomal RNAs were identified using RNAmmer [42]. Other non-coding RNAs were found by searching the genome for corresponding Rfam profiles using INFERNAL [43]. Additional gene prediction analysis and manual functional annotation was performed within the IMG-ER platform.

## Genome properties

The HIMB11 draft genome is 3,098,747 bp long and comprises 34 contigs ranging in size from 454 to 442,822 bp, with an overall GC content of 49.73% (Table 3). Of the 3,237 predicted genes, 3,183 (98.33%) were protein-coding genes, and 54 were RNAs. Most (78%) protein-coding genes were assigned putative functions, while the remaining genes were annotated as hypothetical proteins. The distribution of genes into COG functional categories is presented in Table 4.

**Table 3:** Nucleotide content and gene count levels of the genome

Attribute	Value	% of total^a^
Genome size (bp)	3,098,747	100.00
DNA coding region (bp)	2,812,982	90.78
DNA G+C content (bp)	1,541,077	49.73
Total genes	3,237	100.00
RNA genes	54	1.67
Protein-coding genes	3,183	98.33
Genes in paralog clusters		
Genes assigned to COGs	2,523	77.94
1 or more conserved domains		
2 or more conserved domains		
3 or more conserved domains		
4 or more conserved domains		
Genes with signal peptides	919	28.39
Genes with transmembrane helices	654	20.20
Paralogous groups		

a) The total is based on either the size of the genome in base pairs or the total number of protein coding genes in the annotated genome.

**Table 4:** Number of genes associated with the 25 general COG functional categories

**Code**	**Value**	**%age**^a^	**Description**
J	158	5.6	Translation
A	0	0	RNA processing and modification
K	159	5.7	Transcription
L	104	3.7	Replication, recombination and repair
B	3	0.1	Chromatin structure and dynamics
D	24	0.9	Cell cycle control, mitosis and meiosis
Y	0	0	Nuclear structure
V	22	0.8	Defense mechanisms
T	74	2.6	Signal transduction mechanisms
M	133	4.7	Cell wall/membrane biogenesis
N	40	1.4	Cell motility
Z	0	0	Cytoskeleton
W	0	0	Extracellular structures
U	38	1.4	Intracellular trafficking and secretion
O	118	4.2	Posttranslational modification, protein turnover, chaperones
C	196	7.0	Energy production and conversion
G	165	5.9	Carbohydrate transport and metabolism
E	334	11.9	Amino acid transport and metabolism
F	73	2.6	Nucleotide transport and metabolism
H	157	5.6	Coenzyme transport and metabolism
I	154	5.5	Lipid transport and metabolism
P	112	4.0	Inorganic ion transport and metabolism
Q	123	4.4	Secondary metabolites biosynthesis, transport and catabolism
R	379	13.5	General function prediction only
S	238	8.5	Function unknown
-	714	22.06	Not in COGs

a) The total is based on the total number of protein coding genes in the annotated genome.

## Insights from the Genome Sequence

### Metabolism of HIMB11

**Figure 4:**
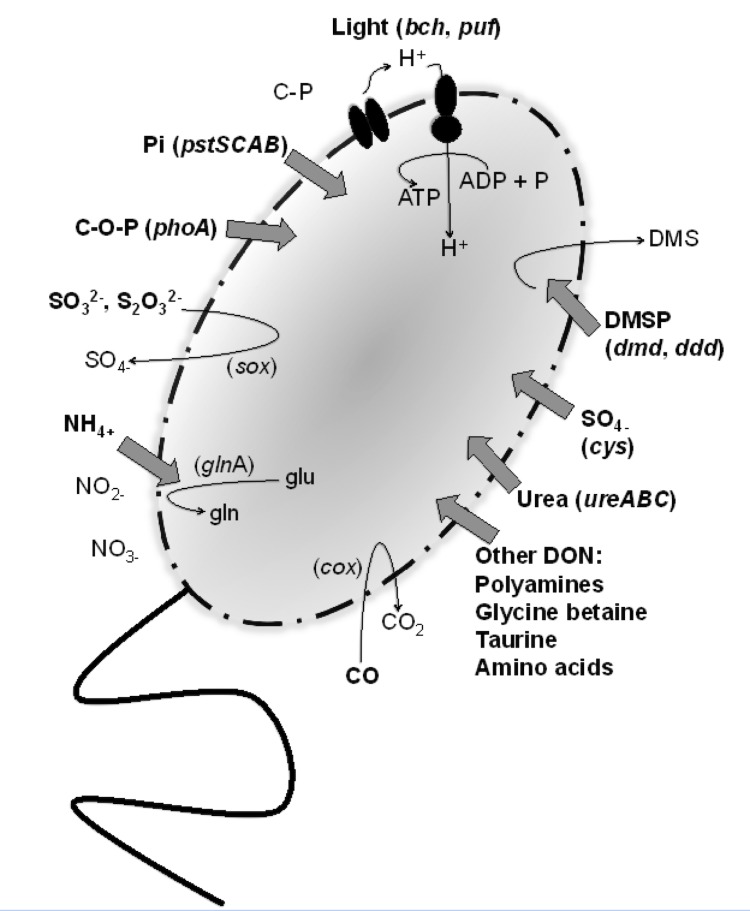
Proposed mechanisms for the acquisition of carbon, nitrogen, phosphorus, sulfur, and energy in HIMB11. Substrates that are hypothesized to be transported and used by HIMB11 are in bold. Genes that designate these mechanisms are indicated. DMSP, dimethylsulfoniopropionate; DMS, dimethylsulfide; DON, dissolved organic nitrogen; C-O-P, phosphoesters; C-P, phosphonates; Pi, phosphate.

Major pathways of carbon, nitrogen, phosphorus, and sulfur acquisition, as well as alternative metabolisms and means of energy acquisition (e.g. light, CO), were annotated based on the presence and absence of key genes involved in these processes. A summary is provided in Figure 4. HIMB11 appears to possess an incomplete glycolysis pathway (*pfkC* and *pgm* are absent), yet it possesses the genes necessary for gluconeogenesis. HIMB11 harbors genes for the Entner-Doudoroff and pentose phosphate pathways, as well as pyruvate carboxylase to perform anaplerotic CO_2_ fixation. HIMB11 does not appear to use inorganic forms of nitrogen other than ammonium, as there are no genes present that are involved in nitrogen fixation, nitrate or nitrite reduction, nitric oxide reduction, nitrous oxide reduction, hydroxylamine oxidation, or nitroalkane denitrification. Instead, HIMB11 is hypothesized to rely solely on reduced and organic nitrogen sources; there are transporters for ammonium (*amtB*) and a variety of other nitrogen-containing substrates (e.g. amino acids, polyamines, glycine betaine, taurine), as well as genes for urease (*ureABC*). Strain HIMB11 possesses a high-affinity phosphate transporter accompanied by regulatory genes (*pstSCAB*, *phoUBR*) and alkaline phosphatase (*phoA*), suggesting that it can utilize both inorganic and organic forms of phosphorus; it does not harbor low-affinity phosphate transport (*pit*A) or the genes for phosphonate utilization (*phnGHIJKLM*).

HIMB11 possesses genes for assimilatory sulfate reduction (*cys*) and for the metabolism of reduced, organic sulfur compounds (e.g. amino acids, DMSP). DMSP is an osmolyte produced by certain phytoplankton, including dinoflagellates and coccolithophores [44,45], and acts as a major source of both carbon and sulfur for marine bacterioplankton in ocean surface waters [46-49]. Roseobacters are frequently abundant during DMSP-producing algal blooms [1], and members of this group have become models for the study of bacterial transformations of DMSP [50]. There are two competing pathways for DMSP degradation: the demethylation pathway that leads to assimilation of sulfur (*dmdA, -B, -C, -D*), and the cleavage pathway that leads to the release of DMS (*dddD*, -*L*, -*P*, -*Q*, *-W*, *-Y*) [51]. DMS is a climate-active gas that has been implicated in the formation of atmospheric-cooling aerosols and clouds. The genome of HIMB11 harbors versions of both sides of the pathway (*dmdA*, -*B*, -*C*, -*D*’ and *dddP*, -*D*).

The HIMB11 genome contains genes that encode for a diverse array of energy acquisition strategies. The presence of the *sox* gene cluster indicates that HIMB11 is putatively capable of oxidizing reduced inorganic sulfur compounds [8] as a mechanism for lithoheterotrophic growth. Additional modes of energy acquisition encoded by the HIMB11 genome include pathways for CO oxidation to CO_2_ via carbon monoxide dehydrogenase (i.e. *cox* operons, including *cox*L, forms I and II) [25,26], degradation of aromatics (i.e. gentisate, benzoate, phenylacetic acid), and bacteriochlorophyll-based anoxygenic photosynthesis. Photosynthetic genes are organized in a photosynthesis gene cluster (PGC) and include genes for the photosynthetic reaction center (*puf* and *puh*), light harvesting complexes, biosynthesis of bacteriochlorphyll *a* and carotenoids, and regulatory factors (*bch* and *crt*). Two conserved regions within the PGC that were identified in a recent study examining the structure and arrangement of PGCs in ten AAnP bacterial genomes of different phylogenies, *bchFNBHLM-LhaA-puhABC* and *crtF-bchCXYZ* [28], were also found to be conserved in the HIMB11 genome. The arrangement of the *puf* genes (*pufQBALMC*) as well as the *puh* genes (*puhABC-hyp-ascF-puhE*) in HIMB11 is very similar to what has been described before for other *Roseobacter* strains [28]. Putative genes containing the sensor domain BLUF (blue-light-utilizing flavin adenine dinucleotide) were also found in HIMB11. BLUF sensor domains have been hypothesized to be involved in a light-dependent regulation of the photosynthesis operon and may enable light sensing for phototrophy [5,52].

## Genome comparisons with other members of the *Roseobacter* clade

**Figure 5:**
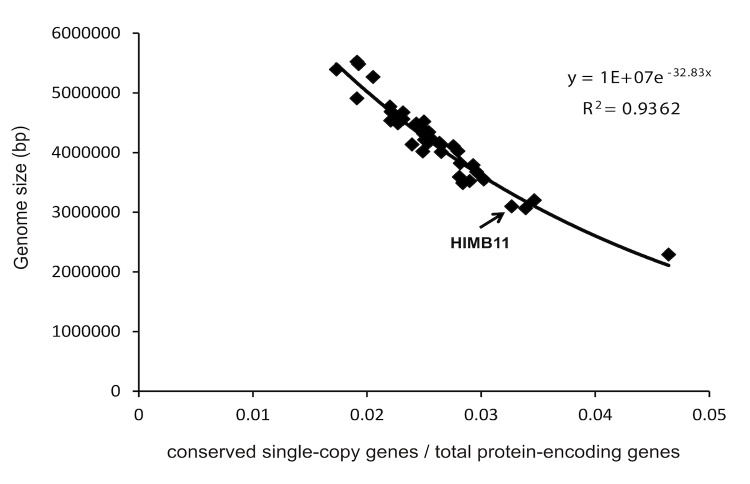
Regression model for strain HIMB11 genome size estimation based on the genomes of 40 cultured *Roseobacter* strains. The x-axis shows the ratio of the number of conserved single-copy genes universally present in fully sequenced *Roseobacter* genomes to the number of predicted protein-encoding genes. The y-axis is the number of nucleotides sequenced. The data were fit to an exponential regression model (R^2^=0.94), and the model was used to estimate the genome size of HIMB11 to be 3.42 Mb.

A regression model was used to estimate the genome size of HIMB11 based on the genomes of 40 *Roseobacter* strains (Figure 5). The model considers the number of nucleotides sequenced versus the ratio of the number of conserved single-copy genes universally present in *Roseobacter* genomes to the number of predicted protein-encoding genes. These data were fit to an exponential regression model (R^2^=0.94), which estimates the genome coverage of the draft HIMB11 genome to be 90.6% and the full genome size to be 3.42 Mb. This is relatively small compared to most cultured *Roseobacter* genomes (median 4.35 Mb) with only one notable exception (*Roseobacter* member HTCC2255, 2.21 Mb).

At the time of this analysis, 35 other *Roseobacter* genomes were publically available in the IMG-ER database (Table 5). The effect of a reduced genome size is readily apparent with respect to the transporter content of the HIMB11 genome: it possesses a highly reduced number of genes devoted to ATP-binding cassette (ABC) transporters and tripartite ATP-independent periplasmic (TRAP) transporters. ABC transporters use energy from ATP hydrolysis to transport a wide range of substrates across the membrane (e.g. ions, amino acids, peptides, sugars). While the 35 public *Roseobacter* genomes contain on average 279 genes involved in ABC transport (171 to 443 per genome), HIMB11 has only 169 genes for ABC transport systems. TRAP transporters are also underrepresented in the HIMB11 genome. These are a large prokaryotic family of solute transporters that contain a substrate binding protein (DctP) and two membrane proteins (DctQ and DctM). By relying on electrochemical ion gradients rather than ATP for transport [53], they mediate the uptake of a number of different substrates (e.g. succinate, malate, fumarate, pyruvate, taurine, ectoine, DMSP). *Roseobacter* genomes contain on average 60 genes devoted to TRAP transporter systems (23 to 135 genes per genome), while the HIMB11 genome harbors 26 TRAP transporter genes.

**Table 5:** Publicly available *Roseobacter* clade genomes use for comparative analysis with strain HIMB11, as of IMG release 3.4.

**Organism name**	**Status**	**Size (bp)**	**Gene Count**	**GC%**
*Citreicella* sp. SE45	Draft	5,523,231	5,499	67
*Dinoroseobacter shibae* DFL-12, DSM 16493	Finished	4,417,868	4,271	66
*Jannaschia* sp. CCS1	Finished	4,404,049	4,339	62
*Loktanella* sp. CCS2	Draft	3,497,325	3,703	55
*Loktanella vestfoldensis* SKA53	Draft	3,063,691	3,117	60
*Maritimibacter alkaliphilus* HTCC2654	Draft	4,529,231	4,763	64
*Oceanibulbus indolifex* HEL-45	Draft	4,105,524	4,208	60
*Oceanicola batsensis* HTCC2597	Draft	4,437,668	4,261	66
*Oceanicola granulosus* HTCC2516	Draft	4,039,111	3,855	70
*Octadecabacter antarcticus* 238	Draft	5,393,715	5,883	55
*Octadecabacter antarcticus* 307	Draft	4,909,025	5,544	55
*Pelagibaca bermudensis* HTCC2601	Draft	5,425,920	5,519	66
*Phaeobacter gallaeciensis* 2.10	Draft	4,157,399	4,017	60
*Phaeobacter gallaeciensis* BS107	Draft	4,232,367	4,136	60
*Rhodobacterales* sp. HTCC2083	Draft	4,018,415	4,226	53
*Rhodobacterales* sp. HTCC2150	Draft	3,582,902	3,713	49
*Rhodobacterales* sp. Y4I	Draft	4,344,244	4,206	64
*Rhodobacterales* sp. HTCC2255	Draft	2,224,475	2,209	37
*Roseobacter denitrificans* OCh 114	Finished	4,331,234	4,201	59
*Roseobacter* sp. AzwK-3b	Draft	4,178,704	4,197	62
*Roseobacter* sp. MED193	Draft	4,652,716	4,605	57
*Roseobacter* sp. SK209-2-6	Draft	4,555,826	4,610	57
*Roseovarius nubinhibens* ISM	Draft	3,668,667	3,605	64
*Roseovarius* sp. 217	Draft	4,762,632	4,823	61
*Roseovarius* sp. TM1035	Draft	4,209,812	4,158	61
*Ruegeria pomeroyi* DSS-3	Finished	4,601,053	4,355	64
*Ruegeria* sp. KLH11	Draft	4,487,498	4,338	58
*Ruegeria* sp. TM1040	Finished	4,153,699	3,964	60
*Sagittula stellata* E-37	Draft	5,262,893	5,121	65
*Silicibacter lacuscaerulensis* ITI-1157	Draft	3,523,710	3,677	63
*Silicibacter* sp. TrichCH4B	Draft	4,689,084	4,814	59
*Sulfitobacter* sp. EE-36	Draft	3,547,243	3,542	60
*Sulfitobacter* sp. GAI101	Draft	4,527,951	4,258	59
*Sulfitobacter* sp. NAS-14.1	Draft	4,002,069	4,026	60
*Thalassiobium* sp. R2A62	Draft	3,487,925	3,744	55

In contrast, drug/metabolite transporters (DMTs), which are another abundant group of transporters found in roseobacters [5], are abundant in HIMB11. DMTs are a ubiquitous superfamily (containing 14 families, six of which are prokaryotic) of drug and metabolite transporters, of which few are functionally characterized [54]. In prokaryotes, most act as pumps for the efflux of drugs and metabolites. On average, individual *Roseobacter* genomes harbor 27 genes for DMTs (19 to 37 per genome). HIMB11 has 33 DMT genes, which is further elevated when normalized to its small genome size. Thus, the reductive trend for ABC and TRAP transporters is reversed in the DMT family of transporters, potentially a result of selective pressure for the efflux of toxins/metabolites.

## Conclusion

HIMB11 represents a member of the *Roseobacter* lineage that is phylogenomically distinct from other cultured, sequenced members of the *Roseobacter* clade. This uniqueness is further supported by its small genome and cell size relative to other members of this group that have been similarly investigated. These characteristics, taken together with the atypical transporter inventories, the presence of many alternative methods of energy acquisition (e.g. CO, light), and the periodic abundance of HIMB11 in Kaneohe Bay, suggest that stain HIMB11 is an opportunist in the environment, persisting on relatively few reduced substrates and alternative energy metabolism until conditions arise that are favorable for rapid growth (e.g. a phytoplankton bloom). Consistent with other members of this lineage is the potential for HIMB11 to play an important role in the cycling of the climatically important gases DMS, CO, and CO_2_, warranting further study in both the laboratory and field.
